# Defining the Dimensions of Diversity to Promote Inclusion in the Digital Era of Health Care: A Lexicon

**DOI:** 10.2196/51980

**Published:** 2024-02-09

**Authors:** Yashoda Sharma, Anindita Saha, Jennifer C Goldsack

**Affiliations:** 1 Digital Medicine Society Boston, MA United States; 2 Center for Devices and Radiological Health US Food and Drug Administration Silver Spring, MD United States

**Keywords:** digital medicine, inclusion, digital health technology/product, digital health, digital technology, health care system, innovation, equity, quality, disparity, digital era, digital access, digital literacy

## Abstract

The pandemic provided a stark reminder of the inequities faced by populations historically marginalized by the health care system and accelerated the adoption of digital health technologies to drive innovation. Digital health technologies’ purported promises to reduce inefficiencies and costs, improve access and health outcomes, and empower patients add a new level of urgency to health equity. As conventional medicine shifts toward digital medicine, we have the opportunity to intentionally develop and deploy digital health technologies with an inclusion focus. The first step is ensuring that the multiple dimensions of diversity are captured. We propose a lexicon that encompasses elements critical for implementing an inclusive approach to advancing health care quality and health services research in the digital era.

## Introduction

### A Lack of Diversity Limits Health for All

As the health care industry undergoes digitization, leaders across the field have the opportunity to develop approaches that can advance access and increase equity and quality. To do this, we need to examine the source of health disparities in the current system: the underrepresentation of specific populations as participants in research and inadequate health care access for all patients. By recent accounting, less than 3% of published genome-wide association studies include data on people of African, Hispanic, or Latin American ancestry, and 86% of clinical trial participants are of European ancestry [1]. This type of exclusion and the accumulation of health disparities as a consequence of inadequate access to care are deeply rooted in systemic and structural racism [2].

The pandemic’s inequitable toll on public health and health care was fueled by knowledge gaps resulting from decades of exclusion and inadequate care for the most vulnerable populations. COVID-19 infection and death rates, one example of public health consequences of health disparities, highlighted the dire consequences of a lack of diversity in research. Groups historically underserved based on the dimensions of race and ethnicity are disproportionately affected and dying at higher rates than their distribution in the population. Additional dimensions of diversity, such as lower socioeconomic status and limited English proficiency, continue to influence health burdens, health outcomes, and overall quality of life [3-5].

Currently, federal policies driving diversity initiatives for patients in health care and participants in research focus on race and ethnicity [6-9] and are intended to encourage and support research that includes more racial and ethnic groups, as well as women. Some progress has been made; however, the focus has been narrow and incomplete. Attention to race, ethnicity, or sex is not sufficient to promote broad health equity. We propose widening the focus of health equity to better capture the many dimensions of diversity. Age, gender, sexual identity, socioeconomic status, educational attainment, physical and cognitive abilities, access to care, and geography also impact care and quality of life.

Social determinants of health (SDOH), that is, the conditions and environment in which people live, work, learn, and play, significantly contribute to health inequities [10]. When overlaid with the intersectionality of race, ethnicity, age, gender, and sexual identity, SDOH expands the categorization for diversity and populations underserved by health care [11]. The All of Us Research Program [12], which was developed to increase representation in biomedical research, expanded the criteria for diversity to reflect this intersectionality and interdependence of factors affecting health. Now, a similar expansion is necessary to account for the many factors that contribute to a lack of digital equity and inclusion, including SDOH [13], digital access and digital and health literacy [14-16], and community lived experiences [17].

### Define Diversity to Promote Inclusivity

The digitization of health care and research has the potential to transform how we care for people and develop new medical products [18,19]. The Digital Medicine Society, in collaboration with the Center for Devices and Radiological Health of the Food and Drug Administration (FDA) and with guidance issued by the FDA [7,8], has been at the forefront of implementing cross-disciplinary approaches to advance the ethical, effective, equitable, and safe use of digital technologies to redefine health care and improve lives by addressing relevant evidentiary, security, ethical, regulatory, and legal issues [20]. It is with this cross-disciplinary, multistakeholder approach that we propose a complete lexicon of dimensions of diversity that must be considered to ensure inclusion in the digital era of health care. This lexicon is intended to support not only efforts to increase diversity and promote inclusion but also to ensure that health disparities are not exacerbated by these new technologies.

The COVID-19 pandemic emphasized 2 elements that are critical for equity in the digitization of health care and research. The first is the dire consequences of health care built on research with limited populations and confounded by the inequities of care caused by decades of structural racism [21]. Limited research with Black/African American and Hispanic people has resulted in less access to safe and effective medications and a higher underlying disease burden, which increased the risk of a more severe COVID-19 prognosis or higher rates of death [22]. The second is a glimpse at the potential of digital health solutions. However, as digital health solutions have accelerated the transformation of health care, the decades-long systemic barriers to health equity continue to hamstring the potential for effective care for all patients. We saw this for telehealth visits, which were higher among those identifying as White and earning at least US $100,000 [23], and low vaccine distribution and adoption due to early reliance on technology [24]. This underuse is a clear example of the need for inclusivity; it is not sufficient to implement technologies and to hope people will access and use them.

An intentional commitment to inclusion is critical during the development and deployment of digital health solutions to facilitate and advance equity. The All of Us Research Program definition of “underrepresented in biomedical research” [12] must be expanded to include digital inclusion to continue to address health disparities centered on demographics, as well as environmental and lifestyle factors.

### New Dimensions of Diversity for Inclusion in the Digital Era of Medicine

Digital health is the umbrella term for the intersection of technology and health care [25]. Digital medicine is the field of evidence-based digital health tools that measure or intervene in the service of health to support the practice of medicine broadly. To advance health equity, we need to focus on inclusion in digital medicine [26].

Inclusion in digital medicine means (1) being cognizant of characteristics of different populations, and (2) tailoring solutions to ensure that digital health products meet the needs of and benefit, all individuals and communities. This entails not just addressing the needs of individuals who face barriers to digital technology use but also addressing the historical, institutional, structural, and discriminatory forces that created and continue to perpetuate the digital divide [27] and health inequality. We combined the dimensions of age, race, ethnicity, education, socioeconomic status, religion, ability, location, gender, sexual preference, language, ability, and digital technology access and literacy and propose a new, expanded lexicon of dimensions in diversity suitable for the digital era of health (Table 1).

**Table 1:** A newly expanded lexicon of dimensions in diversity suitable for the digital era of medicine.

Diversity dimensions	Characteristics
Age^a^	Pediatric and adolescent populations and adults older than 65
Annual household income^a^	Individuals with annual incomes equal to or below 200% of the Federal poverty level
Digital technology access	Communities with limited access to high-speed internet, such as broadband, or access to digital technologies, such as computers and tablets
Digital technology literacy	Individuals or communities not well versed in the use of digital technology (eg, connecting to the internet and Bluetooth pairing)
Disability^a^	Individuals with either a physical or cognitive disability, including visual, auditory, and mobility
Educational attainment^a^	Individuals with less than a high school degree or equivalent and individuals with limited health literacy
Gender identity^a^	Individuals who identify as a gender variant, nonbinary, transgender, or something else
Geography^a^	Individuals who reside in rural or nonmetropolitan areas, individuals residing in areas with limited internet access, and individuals who are homeless
Language	Individuals with limited English proficiency (written or spoken)
Race and ethnicity^a^	Individuals who identify as other than White and non-Hispanic based on their ancestry (eg, African Americans/Black, Asian, Hispanic/Latinx, Native Hawaiian or Pacific Islander, and Middle Eastern or North African)
Cultural practices	Individuals or communities that may abstain from accessing and using digital technologies (eg, some religions discourage the use of technology on certain days)
Sex identified at birth^a^	Individuals who are neither male nor female (eg, intersex)
Sexual orientation^a^	Individuals who identify as asexual, bisexual, gay or lesbian, or something else

^a^Definitions adopted from Mapes et al [12]. The other definitions were developed by members of the Digital Health Measurement Collaborative Community [28].

Digital medicine can prioritize inclusion through a human-centered, fit-for-purpose lens; that is ensuring that digital products do what they claim for all intended users and serve all members of the populations who can benefit from them. A focus on inclusion will capture diverse populations and lead to more equitable solutions and outcomes for health care. This will also address a big challenge for digital health technologies: trust [29]. Trust is important for full participation; full participation leads to health equity—the state in which everyone has an opportunity to attain their highest level of health [30]. Mistrust of health care systems has been growing for years and has been amplified with the COVID-19 pandemic. Digital health product developers have also contributed to this mistrust, especially when people do not see technology designed for them. A clear example is the photoplethysmography (PPG) optical sensors used in many fitness trackers and pulse oximeters. While PPG sensors have been shown to be inaccurate in people with darker pigmentation [31-33], the technology persists in many widely available products. Developers are evolving fitness trackers [34] with PPG sensors to play a bigger role in collecting relevant health data; however, there are few indications that the limitations of this technology for inclusivity are being addressed.

In the digital era of health, digital access and literacy have emerged as defining factors for equity. Access to high-speed internet, reliable and secure Wi-Fi, and tools such as computers, tablets, and smartphones are greatly impacted by socioeconomic status [35]. Digital health literacy, from recognizing the relevance and value of digital health products in your care to having products in your native language, will dictate the type and level of care you receive. There are currently several limitations to inclusion including resource allocation, data and knowledge gaps, and education and training. A multistakeholder approach which includes health care institutions, policymakers, and research and education systems is required to overcome current limitations.

The proposed new complete lexicon for dimensions of diversity for the digital era of health can effectively support intentional approaches to digital health solution design and deployment [28] for inclusivity. The lexicon is designed to support the intersectionality of specific populations and communities that the field of digital health is intended to serve, allowing for some level of customization for each person. The dimensions of diversity complement Richardson et al’s [36] framework for digital health equity, which expanded the National Institute on Minority Health and Health Disparities Research Framework. Our lexicon provides a level of granularity that those developing or deploying (either in clinical care or research) digital health products should sufficiently represent to ensure an inclusive experience. The lexicon has been applied to the digital health measurement market opportunity calculator, which enables digital health measurement product developers to build a business case for incorporating inclusive practices into their research and development processes [37]. Users of the market opportunity calculator select a dimension of diversity, for a health condition, and receive an output estimating the increase in engagement size and potential increase in market value when that product is made with an inclusive lens for that dimension. The lexicon can also be applied to the Partner, Identify, Demonstrate, Access, Report equity framework for behavioral digital health interventions [38] to focus efforts on specific diversity dimensions and define specific actions for each point of the framework. These equity frameworks provide a structure whereby our lexicon can be added for inclusivity. Together these can lead to transformational changes that will advance health equity.

### Insist on Action

Health equity in clinical research was strengthened by the 2023 omnibus spending bill and the FDA’s diversity plan [39] and is projected to continue in the next year. In a recent Deloitte report, health care and life science leaders rank health equity as a top 10 priority [40]. Partnerships across health care, especially health care providers, communities, and digital health product developers will be instrumental in advancing equity and driving inclusion. A level of accountability will be required to demonstrate early progress; the lexicon for the dimensions of diversity can easily be customized in a dashboard [41] to track and inform strategies and resources for more inclusive digital product development and deployment.

The Digital Medicine Society is hosting the Digital Health Measurement Collaborative Community (DATAcc) [28]. Advancing health equity with collaborative communities is a priority at the FDA’s Center for Devices and Radiological Health for addressing health care challenges. DATAcc convenes industry leaders, academic and clinical researchers, patient and community organizations, health systems, and government/regulatory representatives to develop and demonstrate best practices to advance harmonized approaches to speed the use of digital health measurement to improve health outcomes and health equity. Building on the medical device product lifecycle [42], DATAcc created resources to action the expanded dimensions of diversity to drive inclusivity in both digital health technology development and deployment [37].

Recognizing that the efforts to drive inclusion falls on everyone in the health care and health research ecosystems, DATAcc also designed resources to guide clinical care and research teams in assisting patients with understanding complex concepts associated with digital health product use, such as data privacy and security, and end user agreements. With widespread use, validation, and verification inclusive product development and deployment resources can lead to industry-wide adoption and formalization of inclusivity in digital health care. Thereby, building digital medicine on the foundation of inclusion.

## Conclusions

Leaders in health care are at a crucial juncture that will shape the future of digital medicine. We can develop technologies in a way that is more inclusive to ensure that advances in digital medicine are available and used by all populations. We also recognize that inclusive product development is necessary but not sufficient; end users (patients and research participants) need complete information and training on these products so they can be informed. This has to be an ongoing, ever-evolving process that can grow and change with the adoption of new, more complex technologies. The proposed lexicon offers leaders and organizations the granularity they need to demonstrate their health equity efforts. Inclusivity is the key to accelerating health equity so that diverse populations become more integrated and are better served by the health care system.

## Abbreviations

DATAcc: Digital Health Measurement Collaborative Community
FDA: Food and Drug Administration
PPG: photoplethysmography
SDOH: social determinants of health

## Data Availability

Data sharing is not applicable to this article as no data sets were generated or analyzed during this study.
